# Mapping the Dynamic Complexity of Hypertension Management in São Paulo, Brazil

**DOI:** 10.3389/ijph.2025.1608895

**Published:** 2026-01-07

**Authors:** Pei Shan Loo, Anna Socha, Mariana Silveira, Yara Carnevalli Baxter, Álvaro Avezum, Luciano F. Drager, Luiz A Bortolotto, Johannes Boch, Daniel Cobos Munoz

**Affiliations:** 1 Swiss Tropical and Public Health Institute, Allschwil, Switzerland; 2 University of Basel, Basel, Switzerland; 3 System Dynamics Group, University of Bergen, Bergen, Norway; 4 Beneficência Portuguesa de São Paulo, São Paulo, Brazil; 5 Novartis Foundation, São Paulo, Brazil; 6 Sociedade de Cardiologia do Estado de São Paulo, São Paulo, Brazil; 7 Novartis Foundation, Basel, Switzerland

**Keywords:** hypertension, system thinking, causal loop diagram, Brazil, São Paulo

## Abstract

**Objectives:**

Hypertension is a major cardiovascular risk factor in Brazil and globally, requiring effective healthcare system strategies. This study examines how the health system in São Paulo manages hypertension, identifying patterns and connections that influence patient outcomes and resource use.

**Methods:**

Using literature reviews and participatory discussions with experts, we developed a systems map, causal loop diagram, to illustrate dynamic complexity underpinning hypertension management. Thematic analysis of qualitative data informed the model, highlighting key interactions that shape screening, treatment, and long-term care.

**Results:**

The analysis reveals critical dynamics at individual, community, and system levels. Early diagnosis and expanded treatment access improve adherence and reduce complications. However, these improvements also increase the number of patients needing long-term care. This creates a challenge where healthcare gains today can raise future demands if prevention efforts are underfunded.

**Conclusion:**

Understanding these interconnections is crucial for balancing treatment expansion with sustainable prevention strategies. By mapping system-wide challenges, this study offers a framework to help policymakers allocate resources more effectively and strengthen urban health systems. Future research will focus on using simulation modeling to test policy interventions and improve hypertension outcomes.

## Introduction

Hypertension remains a significant public health challenge globally, including in Brazil, with prevalence rates ranging from 32.3% to 53% [1–5]. This condition significantly contributes to ischemic heart disease, heart failure, and strokes, the leading causes of mortality in the country, and also poses a risk for chronic kidney disorders, and other debilitating health issues [4, 6–8]. The escalating costs associated with treatment and complications underscore the urgent need for policymakers to prioritize comprehensive prevention and management strategies [9–11]. However, there remains a gap in understanding how health systems internally adapt to these pressures, particularly in complex urban settings like São Paulo, where resource constraints and competing demands amplify systemic challenges.

Hypertension management in Brazil is influenced by urbanization, lifestyle changes, and disparities in healthcare access, presenting unique challenges for resource allocation and prioritization [12–15]. Addressing these challenges requires a systems-thinking approach to understand dynamics interactions in health systems. [16, 17]. This study applies systems thinking, specifically through causal loop diagrams (CLDs), as a foundational tool to explore these dynamics and conceptualize interventions.

Existing studies using CLDs have primarily focused on localized or individual-level barriers in hypertension management, such as treatment adherence and care retention. For instance, Ehteshami, Cassidy [18], Qin, Li [19], and Ye, Orji [20] identified barriers to hypertension care seeking and treatment adherence. Iwelunmor, Airhihenbuwa [21] applied a CLD to explore factors influencing the uptake of hypertension treatment medications in West Africa, while Krishna and Franciosa [22] examined disparities in hypertension control in the U.S., emphasizing factors like socioeconomic inequities and policy priorities. While these studies provide valuable insights, they largely overlook the internal dynamics of health systems and how they adapt to the growing burden of hypertension. This study addresses this gap by focusing on internal feedback loops, including resource reallocation and intervention scaling, to strengthen health system resilience and sustainability.

As a large urban center with diverse health challenges, São Paulo provides a unique opportunity to explore how health systems navigate the complexities of hypertension management. Using CLDs as a tool, this study lays the groundwork for a future quantitative simulation model by providing a nuanced understanding of systemic interactions shaping hypertension outcomes. By engaging stakeholders through a group model building approach, this research identifies scalable insights for other urban health systems globally, addressing an important knowledge gap in system-level modeling and informing targeted intervention strategies.

## Methods

This study employed qualitative research methods, guided by systems thinking principles, to develop a causal loop diagram (CLD) that maps hypertension dynamics. The methodology included a literature review to establish an evidence base [23, 24], key informant interviews to capture stakeholder insights, and a participatory group model building approach to conceptualize systemic interactions [25–28]. The study followed the SQUIRE 2.0 guidelines [29] to ensure transparency and rigor in reporting.

Systems thinking provides a framework to navigate these complexities by mapping causal pathways, feedback mechanisms, and delays in health system responses [30, 31]. In health systems research, CLDs are increasingly used to visualize and communicate the multi-faceted and interconnected nature of health challenges [23, 24, 32–34], including non-communicable disease dynamics [16, 26, 35–37].

CLDs depict variables as nodes and causal relationships as arrows, illustrating how elements within a system influence one another through reinforcing or balancing feedback loops [38]. CLDs are not definitive proof of causality but serve as complex causal hypotheses, grounded in theoretical understanding, empirical evidence, or stakeholder insights [32, 39, 40]. They also serve as a foundation for computational simulation, where relationships depicted in the CLD can be quantified and analyzed over time, offering insights into potential leverage points for interventions [16, 26, 41].

Group model building, a participatory approach, actively engaged stakeholders through the exchange, assimilation, and integration of their mental models (perspectives and institutional knowledge) into a holistic system-level mapping of the hypertension problem [42–44]. Four virtual group model building workshops were conducted in March and April 2023, using a series of adapted scripts from the group model building literature [43, 45]. These workshops, led by facilitators, explored key questions such as: what is the problem? Whose problem? How did the problem situation originate? What are the underlying factors? How can the problem be tackled? These workshops refined the CLD iteratively, ensuring it aligned with stakeholder perspectives and priorities.

Patients or the public were not involved in the design, conduct, reporting, or dissemination of this research. Stakeholder engagement focused on actors with experience and institutional knowledge in the hypertension and cardiovascular disease (CVD) management within the São Paulo city context. Stakeholders were identified through a combination of literature review and a snowball approach, leveraging discussions with core contacts in São Paulo involved in the CARDIO4Cities project [6, 10, 11]. CARDIO4Cities is an approach focuses on improving cardiovascular population health through quality of care, early access, policy reform, data and digital innovation, intersectoral collaboration, and local ownership [46].

We prioritized local and global hypertension experts. The final group of stakeholders for the CLD development comprised representatives from key institutions, including the Society of Cardiology of the State of São Paulo, Beneficência Portuguesa, Novartis Foundation (global and local representation), Swiss Tropical and Public Health Institute, International Research Center (Hospital Alemão Oswaldo Cruz), and the University of São Paulo. Additionally, expertise was affiliated from the Population Health Research Institute at McMaster University, Canada.

In total, eight experts contributed to the study, representing diverse fields such as clinical management of CVD and hypertension, public health research, health policy, program implementation, and systems dynamics modeling. Their input ensured the CLD incorporated a broad range of perspectives and expertise, reflecting the multifaceted nature of hypertension management in São Paulo and its global relevance.

### Causal Loop Diagram Development Workflow

Initial meetings introduced stakeholders to systems thinking and project goals. These sessions also served as a platform to discuss primary challenges in hypertension management, leading to the identification of “uncontrolled hypertension” as the central indicator for the CLD and a key metric for monitoring intervention impacts. This initial dialogue informed the project’s direction and fostered commitment among the stakeholders.

A preliminary CLD was developed based on existing literature, incorporating insights from a previous situational analysis of hypertension management [10] and findings from a design thinking workshop in São Paulo [47]. Both authors utilized collaborative and interactive workshop from the design thinking process to understand users’ or stakeholders’ needs, generating innovative solutions, prototyping, and testing ideas.

The stakeholders established that the cascade of care for hypertension, being screening, diagnosis, treatment, and control was a useful framework. This was followed by four group model building sessions, where stakeholders explored problem domains and significant drivers in hypertension burden, further refining the CLD with the 11 key interventions. Following a final round of feedback, the consolidated CLD encapsulated the complexities of hypertension management in São Paulo, serving as a shared tool for understanding hypertension dynamics.

## Results

This section outlines the causal loop diagram (CLD) that analyzes the dynamics of hypertension by initially illustrating feedback loops that form the core of the analysis. The structure is designed to progressively build understanding of these dynamics, beginning with foundational feedback loops and gradually integrating additional interdependencies to offer a comprehensive view of the CLD. Each feedback loop, while valuable on its own, might challenge existing beliefs or seem counterintuitive, and is best understood in relation to the whole CLD.

Overall, five balancing feedback loops based on the global and Brazilian literature were identified. These related to screening and diagnosis, treatment uptake, hypertension burden on the health system, preventive measures at the population level, and preventive measures at the individual level. Two reinforcing feedback loops were identified: firstly, related to the management of chronic hypertension leading to a dependency on screening and treatment and reducing resources for prevention; and secondly, the treatment heavy side effect loop which demonstrates how increasing resources for hypertension treatment can lead to an increased need for hypertension treatment.

The results are presented in three main Sections (*Treatment, Screening and Prevention*) with 2 subsections describing the CLD based on the literature, and stakeholders’ perceptions expressed during the interviews and group model building activities. The ordering of the results (*treatment, screening, and prevention*) reflects the stakeholder-driven sequence of discussion during the co-production workshops. Participants first prioritised pressing service-delivery constraints in treatment, then moved upstream to screening bottlenecks, and finally to prevention.

### Treatment Loops

#### Literature-Based CLD

**FIGURE 1:**
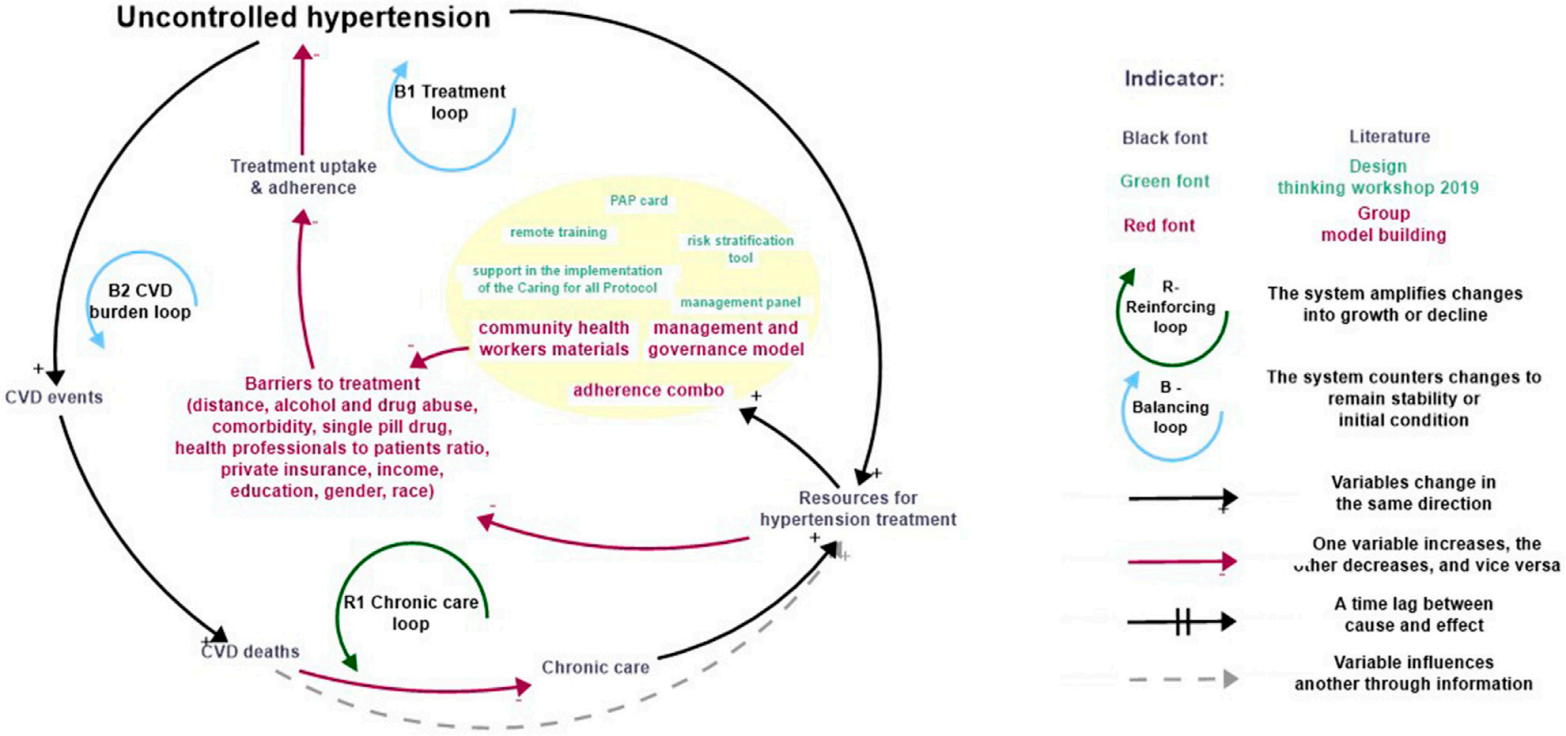
Description of Treatment Loops combining literature, design thinking activities and inputs from the group model building (São Paulo, Brazil, 2025).

Feedback loop B1 the Treatment loop, in Figure 1, shows that an increase in *Uncontrolled hypertension* leads to a rise in *Government budget for hypertension treatment*. This increased allocation meets the growing demand by improving *Uptake and adherence* to hypertension which over time contributes to a reduction in *Uncontrolled hypertension* [48, 49]. The literature [26, 50] substantiates this dynamic, explaining how pressure exerted on governmental and healthcare entities due to the rise in *uncontrolled hypertension* prompts targeted actions in the form of resource allocation to mitigate the burden through treatment.

In addition, *Uncontrolled hypertension* contributes to an increase in *CVD events* over time [7, 8, 51]. This rise in *CVD events*, in turn, results in an escalation of *CVD deaths* [4, 8]. Given that hypertension is a primary risk factor for CVD [3, 48, 49, 52], this mounting burden of CVD inevitably prompts policymakers to allocate more *Resources for hypertension treatment and screening* [26]. This sequence of events, culminating in an amplified response to address hypertension, is visually captured in Feedback loop B2 the CVD burden loop.

Addressing uncontrolled hypertension with increased treatment can paradoxically lead to a rise in the prevalence of the condition. This is because hypertensive patients, now receiving more care, maintain a stable clinical state for longer periods; hence, a drop in CVD events, disability, and deaths [6, 16, 48, 49]. However, since CVD and hypertension are chronic conditions that cannot be completely cured; thus, reducing mortality rates implies that patients with these conditions live longer, necessitating prolonged treatment interventions. This longevity, in turn, triggers an increase in the demand for resources (increases number of patients) over time as the mortality rate reduces while the hypertension onset incident rate remains the same or increases.

This phenomenon is indicative of the “Success to the Successful” archetype, where successful treatment initially leads to cost savings by reducing mortality, in the long term the economic burden may be more significant (i.e., increased healthcare expenditure) due to the chronic nature of these conditions [26]. This dynamic is visually represented in the Feedback loop R1 Chronic care loop in our CLD, highlighting the complexities inherent in health systems responses to chronic diseases such as hypertension.

#### Stakeholder Perceptions

The stakeholders agreed that the Treatment Loops described in the literature taken globally were mostly applicable to São Paulo but identified additional factors influencing treatment adherence and access. Interestingly, treatment adherence (beyond treatment access) emerged as a key area for consideration here; for example, the stakeholders identified a number of patient factors that could influence treatment adherence, such as alcohol and substance abuse, as well as the presence of comorbidities, which were both considered potential obstacles. The stakeholders cited the ratio of health professionals to patients as a factor influencing adherence; to ensure that patients adhere to their treatment plans, the frequency of consultations and the amount of time physicians devoted were regarded as crucial. Additionally, the stakeholders argued that fixed-dose combination therapy (single pill drug) might boost adherence.

For treatment access, stakeholders identified structural and socio-economic barriers, including *distance to health facilities*, *family income*, and *educational level*. They also highlighted that *gender* and *ethnicity* could influence access to care, alongside the availability of *private health insurance*. These contextual factors reflect the complexity of addressing treatment access challenges in São Paulo.

Figure 1 highlights interventions (depicted in yellow) co-created in São Paulo using a design thinking approach in 2019 yellow depicts [53]. These include nine different interventions, which are listed and described in Table 1. When stakeholders were asked to prioritize interventions for inclusion in the simulator, they identified three as most essential. First, the *Management Panels* were considered indispensable for simplifying *hypertension care data*, enabling healthcare providers to better manage care processes and respond effectively to patient needs. Second, the *Adherence Combo* was viewed as an important intervention for improving patient adherence by providing information, raising awareness, and fostering engagement. Lastly, the *Community Health Workers Materials* received unanimous support, with stakeholders emphasizing the importance of enhancing community health workers’ knowledge of *hypertension prevention* and *control* to address gaps in patient education and engagement.

**TABLE 1:** Simplified CARDIO4Cities intervention list [53] (São Paulo, Brazil, 2025).

Intervention	Description
Screening corner (Cantinho cuidando de todos)	Visually engaging physical space in the UBS reception area for opportunistic screening of essential health data (e.g., BP, weight, height, BMI), encouraging self-care and prevention
Health initiatives in schools (heart friends)	Series of training sessions in schools to promote healthy habits and prevent CVDs, integrated with the health at school program
PAP card (Cartão PAP)	A personalized card (now digital) is a “agreed self care plan” to track BP, medications, and appointments, encouraging self-care and treatment adherence
Support in the implementation of the caring for all protocol	Implementation of a municipal policy with workshops and training for clinic staff, providing evidence-based care guidelines for NCD management
Adherence combo (Combo adesão)	Set of tools (e.g., bingo, calendars, medication organizers) to enhance patient adherence to hypertension treatment and lifestyle changes
Remote trainings (Capacitação à distância)	Online courses for health professionals and managers to improve their knowledge and implementation of CVD risk management practices
Management panel	An online dashboard for monitoring the implementation of the caring for all protocol and supporting data-driven decision-making in UBS and regional levels
Management and governance model	Practices and processes to improve healthcare system management, including role definition, data analysis, and regular team collaboration
Risk stratification tool	Tool integrated into the e-saudeSP platform to assess CVD risk and guide prioritization of care for patients based on their risk levels

### Screening Loops

#### Literature-Based CLD

**FIGURE 2:**
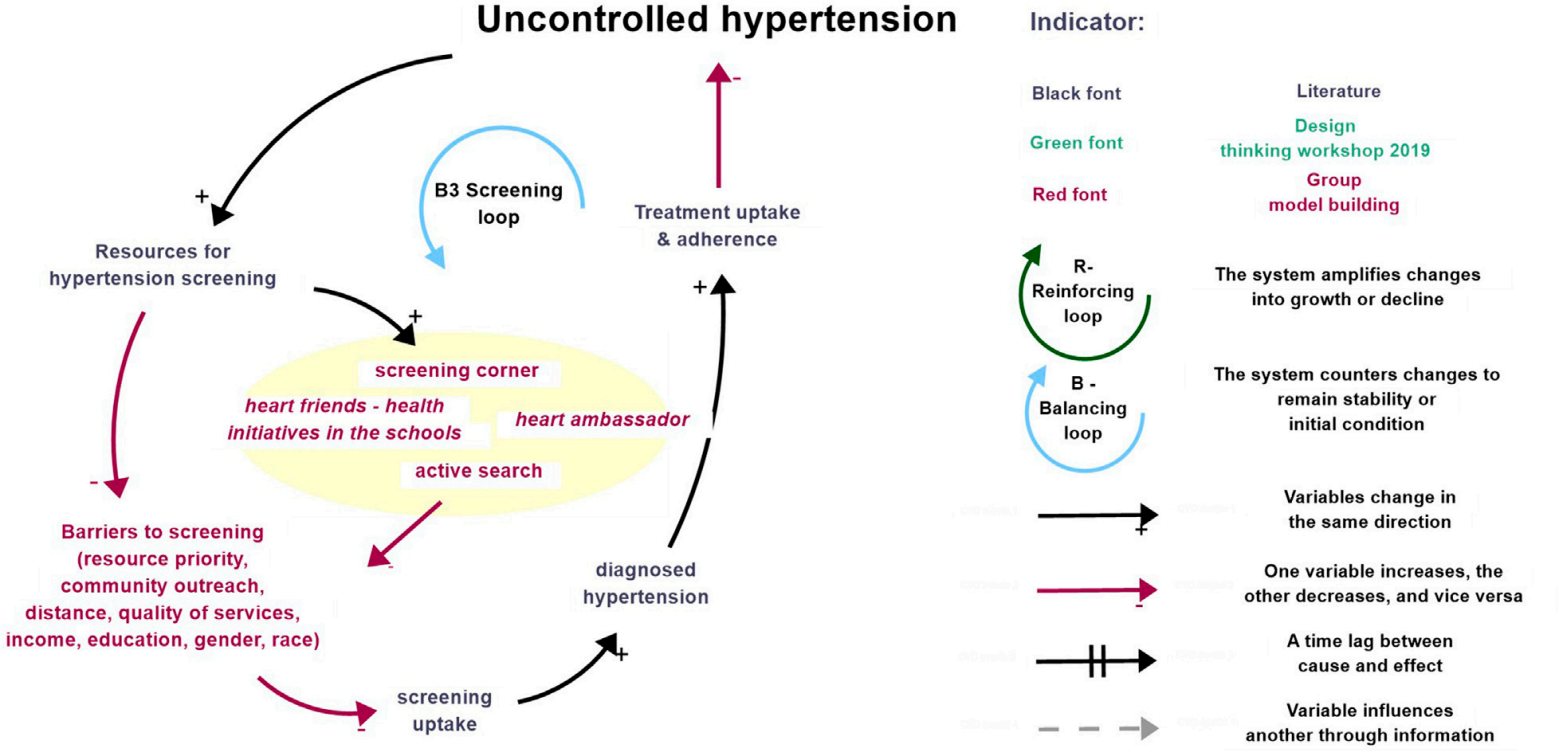
Description of Screening Loops combining literature, design thinking activities and inputs from the group model building (São Paulo, Brazil, 2025).

Feedback Loop B3, also referred to as the “the Screening Loop” in Figure 2, illustrates the hypertension diagnosis dynamics. An increase in *uncontrolled hypertension* prompts a response to boost *resources for hypertension screening*, aimed at early detection and diagnosis. This allocation of resources facilitates an increased *screening uptake*, leading to a rise in *diagnosed hypertension* [54]. As *diagnosed hypertension* increases, it stimulates greater *treatment uptake and adherence* [55]. Over time, this process reduces the initially elevated levels of *uncontrolled hypertension*.

Additionally, Feedback Loop B2 CVD burden (as previously described in Section *Literature-Based CLD*), also influences the allocation of *resources for hypertension diagnosis*. An increase in *CVD burden*, often driven by *uncontrolled hypertension*, pushes policymakers to allocate more resources for screening efforts. This reinforces the *Screening Loop*, illustrating how different feedback loops interact to amplify or mitigate responses in hypertension care.

#### Stakeholder Perceptions

Stakeholders identified several challenges influencing hypertension screening, emphasizing barriers related to health system resources and patient-specific factors. A key concern was the *deprioritization of chronic diseases* like hypertension due to *resource constraints*. Limited *outreach by community health workers* and *geographical distance to healthcare facilities* were seen as significant obstacles. Additionally, the *quality of blood pressure measurements* was noted as a significant health system factor affecting screening accuracy and effectiveness.

Patient-specific barriers also emerged as important considerations. Stakeholders highlighted *socioeconomic factors* such as *family income*, *level of education*, *gender*, and *ethnicity* as determinants of access to screening. For instance, men often face challenges due to *work commitments* and limited flexibility in taking time off, while other demographic groups may face inequities based on their social circumstances.

To address these issues, stakeholders prioritized community-based strategies to expand hypertension screening. They emphasized the *Active Search intervention*, which integrates into the *Caring for All Protocol* training module to enhance *active search* and tracking activities at primary healthcare units (*UBS*). Stakeholders also supported the expansion of the *Screening Corner* initiative at *UBS* facilities, which provides a dedicated space for opportunistic and *self-screening of blood pressure*. This setup was praised for facilitating early detection, improving *data collection*, and encouraging referrals to care, thereby closing gaps between diagnosis and treatment.

### Prevention Loops

#### Literature-Based CLD

**FIGURE 3:**
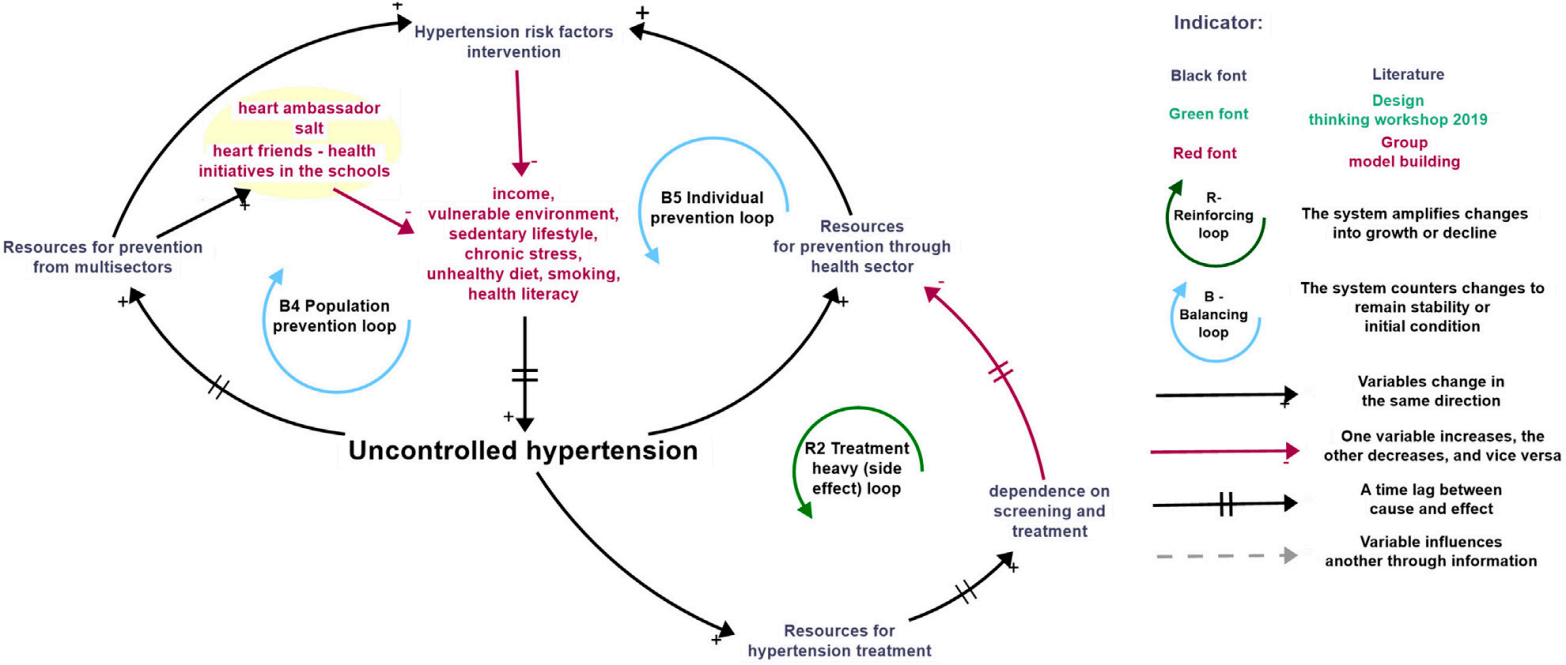
Description of Prevention Loops combining literature, design thinking activities and inputs from the group model building (São Paulo, Brazil, 2025).

Smoking, sedentary lifestyle, chronic stress, and unhealthy diet are established risk factors for hypertension [56–58]. In Figure 3, Feedback Loop B4 on population prevention and Feedback Loop B5 on individual prevention highlight how this risk factors interact with hypertension dynamics. In Feedback Loop B4, societal pressure from uncontrolled hypertension prompts governments to allocate more resources to population-level prevention, including school-based programs, workplace initiatives, and policy reforms. In Feedback Loop B5, increased stress on the health sector leads to greater investment in individual-level prevention measures, such as behavior change interventions. Both types of interventions aim to promote healthier lifestyles, ultimately reducing uncontrolled hypertension [59–63].

A significant delay exists between preventive interventions and their observable impact on hypertension outcomes. This delay, depicted with dashed lines in the diagram, reflects the “balancing process with delay” archetype [64]. Without awareness of this lag, policymakers may reallocate resources to areas with immediate outcomes, such as screening and treatment, undermining the long-term benefits of prevention. This reactive approach risks neglecting prevention, undermining its long-term benefits [64].

Secondly, screening and treatment deliver faster, visible improvements in hypertension and CVD outcomes when implemented together [65–69]. However, this focus on short-term gains reflects the “shifting the burden” archetype [64], where reactive care dominates resources and attention. As a result, the system becomes increasingly reliant on screening and treatment, while preventive interventions are deprioritized (illustrated in Feedback Loop R2 Treatment heavy [side effect] loop).

#### Stakeholder Perceptions

Stakeholders identified a range of risk factors contributing to hypertension, spanning personal lifestyle choices and broader socio-economic and environmental conditions. Socio-economic status was highlighted as a key determinant, with lower-income individuals often confined to vulnerable environments characterized by poor housing, limited healthcare access, and heightened stress. These conditions exacerbate hypertension risk and affect health literacy, which influences behaviors and decisions related to blood pressure management.

Lifestyle factors, particularly diet and sodium intake, were underscored as major contributors. Stakeholders noted that limited access to healthy foods and the prevalence of inexpensive, unhealthy options drive poor dietary choices, including high sodium consumption. They spotlighted Portugal as a successful example, where nationwide policies and public health interventions effectively reduced sodium intake and improved hypertension control [70, 71]. Community vulnerabilities, such as unsafe neighborhoods and economic disadvantage, were also identified as barriers to physical activity and contributors to chronic stress. These factors collectively foster unhealthy coping behaviors, including inadequate physical activity, substance abuse, and poor diets, which increase hypertension risk.

To address these challenges, stakeholders proposed several interventions. Sodium reduction emerged as a priority, reflecting lessons from Portugal’s success. Programs like *Heart Friends at Schools* and *Heart Ambassadors* were endorsed for promoting healthy habits and improving referral mechanisms for hypertension care. These initiatives aim to foster awareness and encourage preventive behaviors in schools and communities, ensuring timely intervention for at-risk individuals. The significant role of community health workers in hypertension prevention was also emphasized. Positioned at the grassroots level, these professionals are uniquely equipped to provide education, screen for hypertension, and facilitate referrals. Their role bridges the gap between healthcare services and community needs, making them pivotal to grassroots prevention efforts.

**FIGURE 4:**
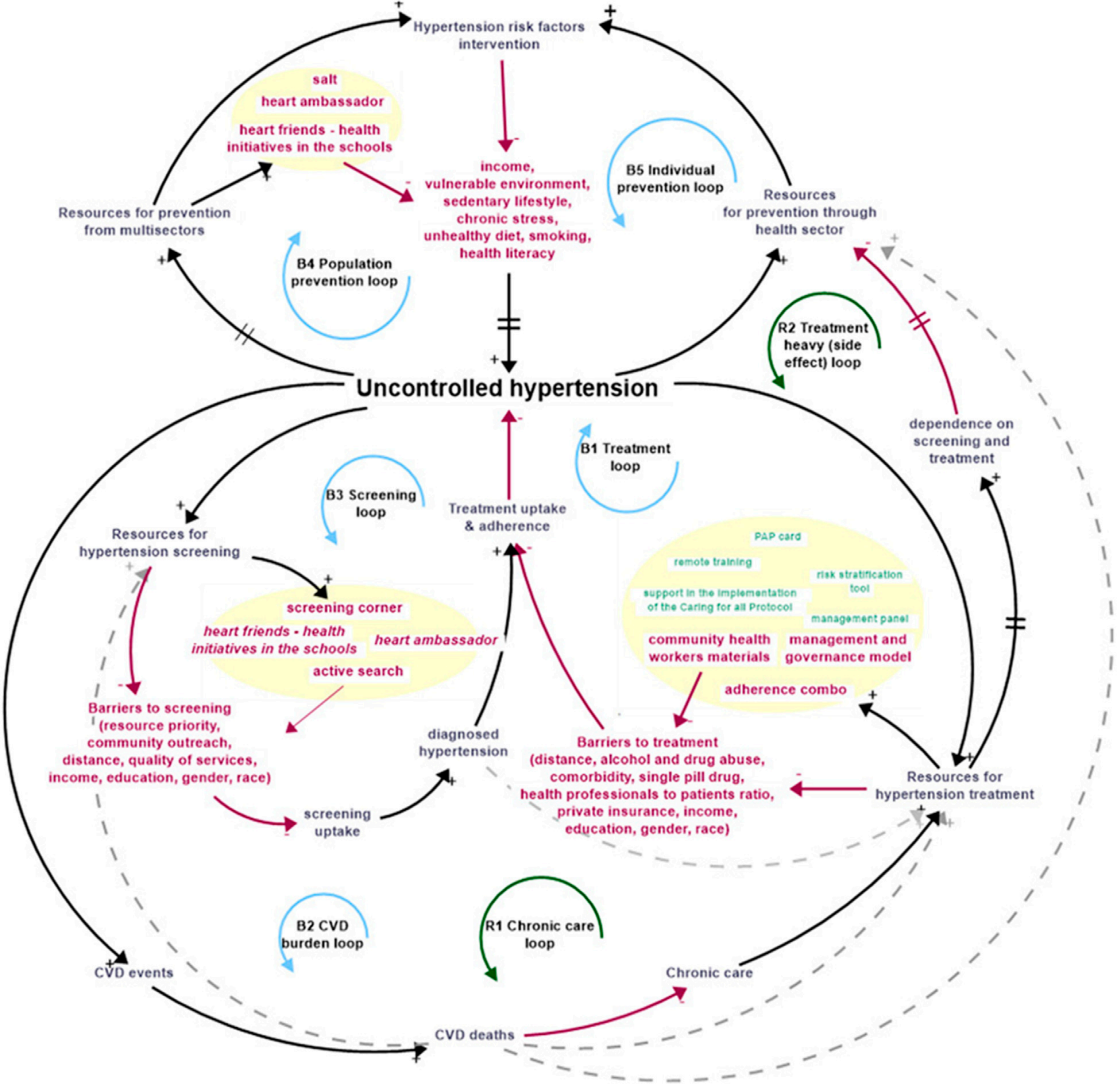
Causal loop diagram with all feedback loops from Table 1 to illustrate hypertension dynamics and management (São Paulo, Brazil, 2025).

Overall, the findings emphasize strategies must address both population-level prevention and individual-level care to effectively reduce uncontrolled hypertension. Figure 4 illustrates the consolidated findings of all the feedback loops while Table 2 outlines each feedback loop.

**TABLE 2:** Simplified feedback loops with literature support (São Paulo, Brazil, 2025).

Loop	Variables (literature)	Literature
B1 treatment loop	Uncontrolled hypertension → resources for hypertension treatment → barriers to treatment → treatment (uptake and adherence) → uncontrolled hypertension	[26, 48–50]
B2 CVD burden loop	Uncontrolled hypertension → CVD events → CVD deaths → chronic care → resources for hypertension screening (and treatment) → barriers to screening and treatment → screening and treatment uptake and adherence → uncontrolled hypertension	[3, 4, 7, 8, 48, 51, 52]
B3 screening loop	Uncontrolled hypertension → resources for hypertension screening → barriers to screening → screening uptake → diagnosed hypertension → treatment uptake and adherence → uncontrolled hypertension	[54, 55]
B4 population prevention	Uncontrolled hypertension → resources for prevention from multisectors → hypertension risk factors intervention → risk factors → uncontrolled hypertension	[59–63]
B5 individual prevention	Uncontrolled hypertension → resources for prevention through health sector → hypertension risk factor intervention → risk factors → uncontrolled hypertension	
R1 chronic care loop	Resources for hypertension treatment → barriers to treatment → treatment (uptake and adherence) → uncontrolled hypertension → CVD events → CVD deaths → chronic care → resources for hypertension treatment	[6, 16, 26, 48, 49]
R2 treatment heavy (side effect) loop	Resources for screening and treatment → barriers to treatment → treatment (uptake and adherence) → uncontrolled hypertension → resources for hypertension treatment → dependence on screening and treatment → resources for prevention through health sector → hypertension risk factor intervention → risk factors → uncontrolled hypertension	[26, 65–69]

## Discussion

This study offers a comprehensive exploration of the dynamic complexity inherent in hypertension management in São Paulo, revealing how multi-level interactions within the health system can shape public health strategies. By integrating literature-based CLD with the insights of diverse stakeholders, our findings illuminate not only the individual and community factors influencing hypertension outcomes, but also the systemic forces that must be addressed to enable sustainable improvements. The CLD developed through this research illustrates that hypertension management is not a series of isolated interventions, but rather a multifaceted process in which individual behaviours, community initiatives, and system-level policies are deeply interdependent.

At the level of the individual, stakeholders emphasized the ongoing challenge of ensuring treatment adherence and promoting healthy lifestyle changes. Strategies such as fixed-dose combination therapies and the use of adherence tools, including the Adherence Combo, emerged as promising approaches for supporting patients in maintaining effective management of their condition. These approaches reflect growing evidence that individualized interventions can be powerful levers for improving outcomes in noncommunicable disease management, as reported in studies across various contexts by Ansah, Islam [17], Iwelunmor, Airhihenbuwa [21], Witter, Zou [72], and Zablith, Diaconu [73].

Moving beyond the clinic, community-level interventions were identified as essential to address the broader social determinants of health. Initiatives like Heart Friends at Schools and Heart Ambassadors were highlighted as effective means of raising awareness, enhancing monitoring, and strengthening referral systems, aligning with best practices seen globally in the promotion of preventive behaviours [74, 75]. These community-based strategies not only foster healthier environments but also empower individuals to take a more active role in their own care.

The study’s systems perspective brings into sharp relief the structural and policy-level barriers that continue to impede progress. Stakeholders underscored the lack of prioritization for chronic disease care within resource-constrained health systems, a challenge echoed in other settings such as Nigeria, where inadequate financing for noncommunicable diseases has similarly constrained public health responses [76, 77]. Overcoming such barriers requires that hypertension management be firmly integrated into broader health policies, with a focus on sustainable resource allocation and the development of workforce capacity. As our findings show, the growing burden of uncontrolled hypertension places mounting strain on healthcare systems, demanding not just incremental increases in resources but more adaptive, resilient approaches to resource management.

Importantly, this study addresses a gap in the existing literature by focusing on how health systems internally adapt to rising demand, rather than solely on risk factors or intervention efficacy. We identified that internal, or endogenous, feedback loops, such as dynamic reallocation of resources, scaling of treatment capacity, and the optimisation of care delivery, are fundamental for maintaining health system resilience and mitigating the chronic disease burden. While much of the literature has documented how external forces such as political priorities and donor funding can shape resource flows Witter, Zou [72], our study demonstrates the crucial role of health systems’ internal dynamics in proactively managing evolving demands [64].

### Policy Implications

The findings have several policy implications. First, expanded screening and diagnosis will necessarily increase the number of individuals requiring treatment, which in turn demands not only greater capacity but also more sustained long-term management of chronic patients. Advances in treatment now enable many individuals to live longer with hypertension, but this places persistent pressure on already stretched healthcare resources. Policymakers must therefore invest strategically in infrastructure, workforce development, and innovative models of care to ensure that all patients receive high-quality, continuous care. Moreover, the need for sustainable resource allocation is paramount. Without careful planning, there is a risk that resources may be shifted away from vital prevention efforts toward immediate treatment needs, undermining the long-term efficacy of public health strategies. Dedicated funding mechanisms, such as ring-fenced budgets for prevention, could help maintain the balance between immediate treatment and sustained prevention activities, supporting the overall goal of long-term hypertension control.

Prevention, while often overlooked in the short term, remains a cornerstone of any effective response to hypertension. Our findings underscore that preventive interventions, whether at the individual or community level, are pivotal for reducing incidence and prevalence, but their benefits typically accrue over longer periods and may be undervalued in resource allocation decisions. Policymakers must recognise and account for these time delays in strategic planning, ensuring continuous investment in prevention even when immediate outcomes are less visible. Sustained support for such initiatives has the potential to yield substantial improvements in population health and to generate cost savings over the long term [59, 70].

A major strength of this study is the integration of design thinking and group model building methodologies. These approaches enabled the generation of actionable, context-specific insights, ensuring that proposed interventions were not only grounded in stakeholder priorities but also responsive to the lived realities of patients and practitioners. By fostering collaboration and joint problem-solving, group model building contributed to a more holistic and accurate mapping of the system’s feedback loops and leverage points, bridging the gap between theoretical models and practical implementation. This participatory, co-production approach has produced a scalable framework that other urban health systems facing similar challenges may find useful.

Nevertheless, certain limitations must be acknowledged. While the primary feedback loops identified in our CLD reflect health system principles that are likely to hold relevance beyond São Paulo, their direct application in other contexts would require adaptation to local infrastructure, demographics, and resource constraints. The study’s reliance on qualitative inputs from a select group of stakeholders in São Paulo may not fully capture the diversity of experiences in all affected populations, particularly those in underrepresented or marginalised communities. To partially mitigate these limitations, we incorporated insights from a 2019 co-authored design thinking initiative involving patients and community stakeholders, providing a complementary evidence base for our qualitative findings [47]. In addition, future research should aim to broaden stakeholder engagement and further contextualise findings.

To address these limitations and build on the current work, the next phase will focus on developing a quantitative simulation model to test and validate the qualitative insights identified here. Such a model would allow for the testing of various policy interventions and support more robust, evidence-based decision-making. Broader stakeholder involvement, including input from the Ministry of Health, patient advocacy groups, and industry partners, will be essential in ensuring both the inclusivity and practical relevance of subsequent research phases.

### Conclusion

In summary, this study demonstrates that mapping the internal dynamics of health systems through participatory systems modeling can reveal critical pathways for both immediate and sustainable improvement in hypertension management. By highlighting the necessity of balancing investment between treatment and prevention, and by showcasing the value of co-production in research, our findings provide a foundation for future quantitative analysis and policy innovation aimed at improving hypertension outcomes in São Paulo and beyond.

## References

[1] Lamelas P Diaz R Orlandini A Avezum A Oliveira G Mattos A Prevalence, Awareness, Treatment and Control of Hypertension in Rural and Urban Communities in Latin American Countries. J Hypertension (2019) 37(9):1813–21. 10.1097/HJH.0000000000002108 30964825

[2] Zhou D Xi B Zhao M Wang L Veeranki SP . Uncontrolled Hypertension Increases Risk of All-Cause and Cardiovascular Disease Mortality in US Adults: The NHANES III Linked Mortality Study. Sci Rep (2018) 8(1):9418. 10.1038/s41598-018-27377-2 29925884 PMC6010458

[3] Nascimento BR Brant LCC Yadgir S Oliveira GMM Roth G Glenn SD Trends in Prevalence, Mortality, and Morbidity Associated with High Systolic Blood Pressure in Brazil from 1990 to 2017: Estimates from the “Global Burden of Disease 2017” (GBD 2017) Study. Popul Health Metrics (2020) 18(1):17. 10.1186/s12963-020-00218-z 32993676 PMC7526365

[4] Malachias M Plavnik FL Machado CA Malta D Scala LCN Fuchs S . 7th Brazilian Guideline of Arterial Hypertension: Chapter 1 - Concept, Epidemiology and Primary Prevention. Arq Bras Cardiol (2016) 107(3 Suppl. 3):1–6. 10.5935/abc.20160151 27819380 PMC5319472

[5] Santiago ERC Diniz ADS Oliveira JS Leal VS Andrade MIS Lira PIC . Prevalence of Systemic Arterial Hypertension and Associated Factors Among Adults from the Semi-Arid Region of Pernambuco, Brazil. Arq Bras Cardiol (2019) 113(4):687–95. 10.5935/abc.20190145 31432978 PMC7020861

[6] Reiker T Des RS Boch J Partha G Venkitachalam L Santana A Population Health Impact and Economic Evaluation of the CARDIO4Cities Approach to Improve Urban Hypertension Management. PLOS Glob Public Health (2023) 3(4):e0001480. 10.1371/journal.pgph.0001480 37040342 PMC10089359

[7] Hossain A Ahsan GU Hossain MZ Hossain MA Sultana ZZ Arefin A A Prospective Longitudinal Study with Treated Hypertensive Patients in Northern Bangladesh (PREDIcT-HTN) to Understand Uncontrolled Hypertension and Adverse Clinical Events: A Protocol for 5-Years Follow-Up. Plos One (2022) 17(5):e0269240. 10.1371/journal.pone.0269240 35639707 PMC9154182

[8] Silva TLN Klein CH Nogueira AR Salis LHA Silva NAS Bloch KV . Cardiovascular Mortality Among a Cohort of Hypertensive and Normotensives in Rio De Janeiro - Brazil - 1991–2009. BMC Public Health (2015) 15(1):623. 10.1186/s12889-015-1999-4 26152148 PMC4495630

[9] Piccini RX Facchini LA Tomasi E Siqueira FV Silveira DS Thumé E Promoção, Prevenção E Cuidado Da Hipertensão Arterial No Brasil. Revista De Saúde Pública (2012) 46(3):543–50. 10.1590/s0034-89102012005000027 22510974

[10] Palmeirim MS Baxter YC Silveira M Maggion RV Aquino B Avezum Á Situational Analysis of Hypertension Management at Primary Health Care Level in São Paulo, Brazil: Population, Healthcare Professional and Health System Perspectives. BMC Health Serv Res (2024) 24(1):668. 10.1186/s12913-024-10978-1 38807206 PMC11134720

[11] Boch J Venkitachalam L Santana A Jones O Reiker T Rosiers SD Implementing a Multisector Public-Private Partnership to Improve Urban Hypertension Management in Low-and Middle-Income Countries. BMC Public Health (2022) 22(1):2379. 10.1186/s12889-022-14833-y 36536360 PMC9761621

[12] Baldisserotto J Kopittke L Nedel FB Takeda SP Mendonça CS Sirena SA Socio-Demographic Caracteristics and Prevalence of Risk Factors in a Hypertensive and Diabetics Population: A Cross-Sectional Study in Primary Health Care in Brazil. BMC Public Health (2016) 16(1):573. 10.1186/s12889-016-3230-7 27422747 PMC4946130

[13] Miraglia JL Mafra ACCN Monteiro CN Borges LM . The Variation of the Burden of Hypertension and Diabetes in Two Large Districts of the City of São Paulo, Brazil, Based on Primary Health Care Routinely-Collected Data. Plos One (2019) 14(3):e0213998. 10.1371/journal.pone.0213998 30875401 PMC6420009

[14] Moura EC Pacheco-Santos LM Peters LR Serruya SJ Guimarães R . Research on Chronic Noncommunicable Diseases in Brazil: Meeting the Challenges of Epidemiologic Transition. Revista Panamericana De Salud Pública (2012) 31(3):240–5. 10.1590/s1020-49892012000300009 22569699

[15] Lima JRA Campanati SP Cunha ÍÍBR Rosa FRPAC Loureiro LS Barth BT The Enormous Cost of Obesity: Analysis of Public Costs in a Basic Health Unit in Minas Gerais, Brazil. Res Soc Development (2023) 12(5):e1112541383. 10.33448/rsd-v12i5.41383

[16] Peng CQ Lawson KD Heffernan M McDonnell G Liew D Lybrand S Gazing Through Time and Beyond the Health Sector: Insights from a System Dynamics Model of Cardiovascular Disease in Australia. PLOS ONE (2021) 16(9):e0257760. 10.1371/journal.pone.0257760 34591888 PMC8483334

[17] Ansah J Islam AM Koh V Ly V Kol H Matchar DB Systems Modelling as an Approach for Understanding and Building Consensus on Non-Communicable Diseases (NCD) Management in Cambodia. BMC Health Serv Res (2019) 19(1):2. 10.1186/s12913-018-3830-2 30606199 PMC6318956

[18] Ehteshami F Cassidy R Tediosi F Fink G Cobos Muñoz D . Combining Theory-Driven Realist Approach and Systems Thinking to Unpack Complexity of Type 2 Diabetes and Hypertension Management in Low and Middle-Income Countries: Protocol for a Realist Review. Systems (2024) 12(1):16. 10.3390/systems12010016

[19] Qin T Li X Qiao K Bai X Gu M Wang Y . Utilizing Group Model Building to Identify Barriers and Facilitators of Hypertension Management in Primary Health Care, China. Risk Management Healthc Policy (2024) 17:1227–37. 10.2147/RMHP.S454748 38765783 PMC11100508

[20] Ye J Orji IA Birkett MA Hirschhorn LR Walunas TL Smith JD Community-Based Participatory Research and System Dynamics Modeling for Improving Retention in Hypertension Care. JAMA Netw Open (2024) 7(8):e2430213. 10.1001/jamanetworkopen.2024.30213 39190307 PMC11350485

[21] Iwelunmor J Airhihenbuwa CO Cooper R Tayo B Plange-Rhule J Adanu R Prevalence, Determinants and Systems-Thinking Approaches to Optimal Hypertension Control in West Africa. Glob Health (2014) 10:42. 10.1186/1744-8603-10-42 24886649 PMC4046625

[22] Krishna K Franciosa M . Addressing Hypertension Disparities via Systems Dynamics: Insights from Community Health Connections. Cureus (2024) 16(9):e68763. 10.7759/cureus.68763 39371701 PMC11456158

[23] Baugh Littlejohns L Baum F Lawless A Freeman T . The Value of a Causal Loop Diagram in Exploring the Complex Interplay of Factors that Influence Health Promotion in a Multisectoral Health System in Australia. Health Res Policy Syst (2018) 16(1):126. 10.1186/s12961-018-0394-x 30594203 PMC6310960

[24] Beaulieu E Spanjaart A Roes A Rachet B Dalle S Kersten MJ Health-Related Quality of Life in Cancer Immunotherapy: A Systematic Perspective, Using Causal Loop Diagrams. Qual Life Res (2022) 31(8):2357–66. 10.1007/s11136-022-03110-5 35298735 PMC8929267

[25] Allender S Owen B Kuhlberg J Lowe J Nagorcka-Smith P Whelan J A Community Based Systems Diagram of Obesity Causes. PloS One (2015) 10(7):e0129683. 10.1371/journal.pone.0129683 26153893 PMC4496094

[26] Ansah JP Matchar DB Koh V Schoenenberger L . Mapping the Dynamic Complexity of Chronic Disease Care in Singapore: Using Group Model Building in Knowledge Elicitation. Syst Res Behav Sci (2018) 35(6):759–75. 10.1002/sres.2517

[27] Langellier BA Kuhlberg JA Ballard EA Slesinski SC Stankov I Gouveia N Using Community-Based System Dynamics Modeling to Understand the Complex Systems that Influence Health in Cities: The SALURBAL Study. Health and Place (2019) 60:102215. 10.1016/j.healthplace.2019.102215 31586769 PMC6919340

[28] Morais LMO Kuhlberg J Ballard E Indvik K Rocha SC Sales DM Promoting Knowledge to Policy Translation for Urban Health Using Community-Based System Dynamics in Brazil. Health Res Policy Syst (2021) 19(1):53. 10.1186/s12961-020-00663-0 33794907 PMC8015032

[29] Ogrinc G Davies L Goodman D Batalden P Davidoff F Stevens D . SQUIRE 2.0 (Standards for Quality Improvement Reporting Excellence): Revised Publication Guidelines from a Detailed Consensus Process. J Nurs Care Qual (2016) 31(1):1–8. 10.1097/NCQ.0000000000000153 26429125 PMC5411027

[30] Sterman J Business Dynamics, c2000. Irwin/McGraw-Hill (2010).

[31] Fone D Hollinghurst S Temple M Round A Lester N Weightman A Systematic Review of the Use and Value of Computer Simulation Modelling in Population Health and Health Care Delivery. J Public Health (2003) 25(4):325–35. 10.1093/pubmed/fdg075 14747592

[32] Cassidy R Borghi J Semwanga AR Binyaruka P Singh N Blanchet K . How to Do (Or Not to Do) Using Causal Loop Diagrams for Health System Research in Low and Middle-Income Settings. Health Policy Plann (2022) 37(10):1328–36. 10.1093/heapol/czac064 35921232 PMC9661310

[33] Wang Y Hu B Zhao Y Kuang G Zhao Y Liu Q Applications of System Dynamics Models in Chronic Disease Prevention: A Systematic Review. Prev Chronic Dis (2021) 18:E103. 10.5888/pcd18.210175 34941481 PMC8718124

[34] Currie DJ Smith C Jagals P . The Application of System Dynamics Modelling to Environmental Health Decision-Making and Policy-a Scoping Review. BMC Public Health (2018) 18:1–11. 10.1186/s12889-018-5318-8 29587701 PMC5870520

[35] Forrester J . Industrial Dynamics. MA: MIT Press Cambridge (1961). [Google Scholar].

[36] Richardson GP . Core of System Dynamics. In: System Dynamics: Theory and Applications (2020). p. 11–20.

[37] Kenealy T Rees D Sheridan N Moffitt A Tibby S Homer J . A ‘Whole of System’Approach to Compare Options for CVD Interventions in Counties Manukau. Aust New Zealand J Public Health (2012) 36(3):263–8. 10.1111/j.1753-6405.2011.00812.x 22672033

[38] Richardson GP Pugh IIIAL . Introduction to System Dynamics Modeling with DYNAMO. J Oper Res Soc (1997) 48(11):1146. 10.1038/sj.jors.2600961

[39] Carey G Malbon E Carey N Joyce A Crammond BR Carey AL . Systems Science and Systems Thinking for Public Health: A Systematic Review of the Field. BMJ Open (2015) 5(12):e009002. 10.1136/bmjopen-2015-009002 26719314 PMC4710830

[40] Crielaard L Uleman JF Châtel BDL Epskamp S Sloot PMA Quax R . Refining the Causal Loop Diagram: A Tutorial for Maximizing the Contribution of Domain Expertise in Computational System Dynamics Modeling. Psychol Methods (2024) 29(1):169–201. 10.1037/met0000484 35549316

[41] O’Halloran SA Hayward J Valdivia Cabrera M Felmingham T Fraser P Needham C The Common Drivers of Children and Young People’s Health and Wellbeing Across 13 Local Government Areas: A Systems View. BMC Public Health (2024) 24(1):847. 10.1186/s12889-024-18354-8 38504205 PMC10949822

[42] Atkinson J O’Donnell E Wiggers J McDonnell G Mitchell J Freebairn L Dynamic Simulation Modelling of Policy Responses to Reduce Alcohol-Related Harms: Rationale and Procedure for a Participatory Approach. Public Health Res and Pract (2017) 27(1):2711707. 10.17061/phrp2711707 28243673

[43] Andersen DF Richardson GP . Scripts for Group Model Building. Syst Dyn Rev The J Syst Dyn Soc (1997) 13(2):107–29. 10.1002/(sici)1099-1727(199722)13:2<107::aid-sdr120>3.3.co;2-z

[44] Hovmand PS Andersen DF Rouwette E Richardson GP Rux K Calhoun A . Group Model‐Building ‘Scripts’ as a Collaborative Planning Tool. Syst Res Behav Sci (2012) 29(2):179–93. 10.1002/sres.2105

[45] Hovmand P Rouwette E Andersen D Richardson G Calhoun A Rux K Scriptapedia: A Handbook of Scripts for Developing Structured Group Model Building Sessions (2011).

[46] Aerts A Boufford JI . A New Whole-of-City Strategy for Addressing Cardiovascular Population Health. Cities and Health (2023) 7(3):296–302. 10.1080/23748834.2021.1979774

[47] Jarrett C Baxter YC Boch J Carrasco C Cobos Muñoz D Mauro Dib K Deconstructing Design Thinking as a Tool for the Implementation of a Population Health Initiative. Health Res Policy Syst (2022) 20(1):91. 10.1186/s12961-022-00892-5 35986365 PMC9389775

[48] Ettehad D Emdin CA Kiran A Anderson S Callender T Emberson J Blood Pressure Lowering for Prevention of Cardiovascular Disease and Death: A Systematic Review and Meta-Analysis. The Lancet (2016) 387(10022):957–67. 10.1016/S0140-6736(15)01225-8 26724178

[49] Bundy JD Li C Stuchlik P Bu X Kelly TN Mills KT Systolic Blood Pressure Reduction and Risk of Cardiovascular Disease and Mortality. Jama Cardiol (2017) 2(7):775–781. 10.1001/jamacardio.2017.1421 28564682 PMC5710614

[50] Homer J Milstein B Wile K Pratibhu P Farris R Orenstein DR . Modeling the Local Dynamics of Cardiovascular Health: Risk Factors, Context, and Capacity. Prev Chronic Dis (2008) 5(2):A63. 18341798 PMC2396963

[51] Salaroli LB Cattafesta M Petarli GB Ribeiro SAV Soares ACO Zandonade E Prevalence and Factors Associated with Arterial Hypertension in a Brazilian Rural Working Population. Clinics (2020) 75:e1603. 10.6061/clinics/2020/e1603 32785573 PMC7410352

[52] Santo AH Puech-Leão P Krutman M . Trends in Abdominal Aortic Aneurysm-Related Mortality in Brazil, 2000-2016: A Multiple-Cause-of-Death Study. Clinics (2021) 76:e2388. 10.6061/clinics/2021/e2388 33503194 PMC7798134

[53] Avezum Á Drager LF Reiker T Bigoni A Leonel LP Abreu A An Intersectoral Approach to Hypertension Care: Solutions for Improving Blood Pressure Control in São Paulo, Brazil. Am J Hypertens (2024) 37(5):366–78. 10.1093/ajh/hpae005 38214400 PMC11016842

[54] Al-Mohaissen MA Al-Obaid Q AlGhamdi WA Al-Alyani H Dahman SM Al-Wahhabi NA Impact of the 2017 American College of Cardiology/American Heart Association Hypertension Guideline on the Prevalence of Hypertension in Young Saudi Women. East Mediterr Health J (2020) 26(04):426–34. 10.26719/emhj.19.080 32338361

[55] Wachsmuth J Sambharia M Griffin BR Swee M Reisinger HS Lund BC The Prevalence and Treatment of Hypertension in Veterans Health Administration, Assessing the Impact of the Updated Clinical Guidelines. J Hypertens (2023) 41(6):995–1002. 10.1097/HJH.0000000000003424 37071434 PMC10158602

[56] Ndanuko R Tapsell LC Charlton KE Neale EP Batterham M . Dietary Patterns and Blood Pressure in Adults: A Systematic Review and Meta-Analysis of Randomized Controlled Trials. Adv Nutr (2016) 7(1):76–89. 10.3945/an.115.009753 26773016 PMC4717885

[57] Shimbo D . Dietary and Lifestyle Factors in Hypertension. J Hum Hypertens (2016) 30(10):571–2. 10.1038/jhh.2016.57 27600029

[58] Theodoridis X Chourdakis M Chrysoula L Chroni V Tirodimos I Δίπλα Κ Adherence to the DASH Diet and Risk of Hypertension: A Systematic Review and Meta-Analysis. Nutrients (2023) 15(14):3261. 10.3390/nu15143261 37513679 PMC10383418

[59] Honeycutt AA Wile K Dove C Hawkins J Orenstein D . Strategic Planning for Chronic Disease Prevention in Rural America. J Public Health Management Pract (2015) 21(4):392–9. 10.1097/PHH.0000000000000062 25084535

[60] Knowledge AD Kasulkar A Gupta M . Attitude and Practice of Lifestyle Modifications Among Hypertensive Patients Visiting a Tertiary Care Hospital in Central India. Int J Hum Health Sci (Ijhhs) (2024) 8(2):140–146. 10.31344/ijhhs.v8i2.634

[61] Arena R Laddu D Severin R Hall G Bond S , HL-PIVOT Network. Healthy Living and Social Justice. J Cardiopulmonary Rehabil Prev (2021) 41(3):E5–E6. 10.1097/HCR.0000000000000612 33907073

[62] Ayodapo AO Olukokun TA . Lifestyle Counselling and Behavioural Change: Role Among Adult Hypertensives in a Rural Tertiary Institution. South Afr Fam Pract (2019) 61(3):91–6. 10.4102/safp.v61i3.4979

[63] Yang S Yu B Liao K Qiao X Fan Y Li M Effectiveness of a Socioecological Model-Guided, Smart Device-Based, Self-Management-Oriented Lifestyle Intervention in Community Residents: Protocol for a Cluster-Randomized Controlled Trial. BMC Public Health (2024) 24(1):32. 10.1186/s12889-023-17073-w 38166669 PMC10763380

[64] Ansah JP Inn RLH Ahmad S . An Evaluation of the Impact of Aggressive Hypertension, Diabetes and Smoking Cessation Management on CVD Outcomes at the Population Level: A Dynamic Simulation Analysis. BMC Public Health (2019) 19(1):1105. 10.1186/s12889-019-7429-2 31412830 PMC6694535

[65] Diaz R Behr JG Britton BS . Estimating Cost Adjustments Required to Accomplish Target Savings in Chronic Disease Management Interventions: A Simulation Study. Simulation (2015) 91(7):599–614. 10.1177/0037549715584618

[66] Jones AP Homer JB Murphy DL Essien JD Milstein B Seville DA . Understanding Diabetes Population Dynamics Through Simulation Modeling and Experimentation. Am Journal Public Health (2006) 96(3):488–94. 10.2105/AJPH.2005.063529 16449587 PMC1470507

[67] Kang H Nembhard HB Ghahramani N Curry W . A System Dynamics Approach to Planning and Evaluating Interventions for Chronic Disease Management. J Operational Research Society (2018) 69(7):987–1005. 10.1057/s41274-017-0279-3

[68] Milstein B Jones A Homer JB Murphy D Essien J Seville D . Peer Reviewed: Charting Plausible Futures for Diabetes Prevalence in the United States: A Role for System Dynamics Simulation Modeling. Preventing Chronic Disease (2007) 4(3). PMC1955415 17572956

[69] Yarnoff B Bradley C Honeycutt AA Soler RE Orenstein D Amoozegar J Peer Reviewed: Estimating the Relative Impact of Clinical and Preventive Community-Based Interventions: An Example Based on the Community Transformation Grant Program. Preventing Chronic Dis (2019) 16:E134. 10.5888/pcd16.190061 PMC6638589 31274409

[70] Bibbins‐Domingo K Chertow GM Coxson PG Moran AE Lightwood J Pletcher MJ Projected Effect of Dietary Salt Reductions on Future Cardiovascular Disease. New Engl J Med (2010) 362(7):590–9. 10.1056/NEJMoa0907355 20089957 PMC3066566

[71] He FJ MacGregor GA . A Comprehensive Review on Salt and Health and Current Experience of Worldwide Salt Reduction Programmes. J Human Hypertension (2009) 23(6):363–84. 10.1038/jhh.2008.144 19110538

[72] Witter S Zou G Diaconu K Senesi RG Idriss A Walley J Opportunities and Challenges for Delivering Non-Communicable Disease Management and Services in Fragile and Post-Conflict Settings: Perceptions of Policy-Makers and Health Providers in Sierra Leone. Conflict and Health (2020) 14:1–14. 10.1186/s13031-019-0248-3 31921333 PMC6945746

[73] Zablith N Diaconu K Naja F El Koussa M Loffreda G Bou-Orm I Dynamics of Non-Communicable Disease Prevention, Diagnosis and Control in Lebanon, a Fragile Setting. Conflict and Health (2021) 15:1–13. 10.1186/s13031-020-00337-2 33430916 PMC7802297

[74] Imamatsu Y Tadaka E . Factors Associated with Competence in Preventing Non-Communicable Diseases Among Community Health Workers in Japan (2023).

[75] Caprara G . Mediterranean-Type Dietary Pattern and Physical Activity: The Winning Combination to Counteract the Rising Burden of Non-Communicable Diseases (NCDs). Nutrients (2021) 13(2):429. 10.3390/nu13020429 33525638 PMC7910909

[76] Ogungbe O Abasilim C Huffman MD Ojji D None N . Improving Hypertension Control in Nigeria: Early Policy Implications from the Hypertension Treatment in Nigeria Program. Glob Health Res Policy (2024) 9(1):26. 10.1186/s41256-024-00368-9 39010244 PMC11247806

[77] Orji IA Baldridge AS Omitiran K Guo M Ajisegiri WS Ojo TM Capacity and Site Readiness for Hypertension Control Program Implementation in the Federal Capital Territory of Nigeria: A Cross-Sectional Study. BMC Health Serv Res (2021) 21(1):322. 10.1186/s12913-021-06320-8 33836719 PMC8034094

